# Predicting stroke occurrences: a stacked machine learning approach with feature selection and data preprocessing

**DOI:** 10.1186/s12859-024-05866-8

**Published:** 2024-10-15

**Authors:** Pritam Chakraborty, Anjan Bandyopadhyay, Preeti Padma Sahu, Aniket Burman, Saurav Mallik, Najah Alsubaie, Mohamed Abbas, Mohammed S. Alqahtani, Ben Othman Soufiene

**Affiliations:** 1 School of computer engineering, KIIT University, Patia, Bhubaneswar, Odisha 751024 India; 2 Department of Environmental Health, Harvard T H Chan School of public Health, 677 Harrington Avenue, Boston, MA 02115 USA; 3 Department of Computer Sciences, College of Computer and Information Sciences, Princess Nourah bint Abdulrahman University, P.O. Box 84428, 11671 Riyadh, Saudi Arabia; 4 Electrical Engineering Department, College of Engineering, King Khalid University, 61421 Abha, Saudi Arabia; 5 Radiological Sciences Department, College of Applied Medical Sciences, King Khalid University, 61421 Abha, Saudi Arabia; 6 BioImaging Unit, Space Research Centre, University of Leicester, Michael Atiyah Building, Leicester, LE1 7RH UK; 7 PRINCE Laboratory Research, ISITcom, Hammam Sousse, University of Sousse, Sousse, Tunisia

**Keywords:** Stroke prediction, Machine learning, Principal component analysis (PCA), Stacking ensemble, Healthcare analytics, Predictive modeling, Class imbalance, Feature selection, Early intervention

## Abstract

Stroke prediction remains a critical area of research in healthcare, aiming to enhance early intervention and patient care strategies. This study investigates the efficacy of machine learning techniques, particularly principal component analysis (PCA) and a stacking ensemble method, for predicting stroke occurrences based on demographic, clinical, and lifestyle factors. We systematically varied PCA components and implemented a stacking model comprising random forest, decision tree, and K-nearest neighbors (KNN).Our findings demonstrate that setting PCA components to 16 optimally enhanced predictive accuracy, achieving a remarkable 98.6% accuracy in stroke prediction. Evaluation metrics underscored the robustness of our approach in handling class imbalance and improving model performance, also comparative analyses against traditional machine learning algorithms such as SVM, logistic regression, and Naive Bayes highlighted the superiority of our proposed method.

## Introduction

The global population’s growth has coincided with a concerning surge in cases of brain strokes, leading to a notable increase in annual fatalities by 2023. With the number of stroke-related deaths on the rise, the imperative to address this crisis has become increasingly urgent. This alarming trend has propelled stroke research to the forefront of medical exploration.

Machine learning algorithms have shown promise in revolutionizing stroke prediction by analyzing extensive datasets encompassing demographic information, medical histories, and physiological markers like age, blood pressure, and glucose levels [1, 2]. However, the deployment of these algorithms in clinical settings presents challenges that must be addressed. One significant concern is the potential bias embedded within training data, which can lead to skewed predictions and inequitable healthcare outcomes [3]. Biases may arise from incomplete or unrepresentative datasets, socioeconomic factors, or disparities in healthcare access.

To mitigate these challenges, ensemble learning methods such as stacking have emerged as a robust approach.Our model which involves stacking integrates predictions from the base classifiers-random forest, Decision Tree and final estimator which is KNN-to enhance predictive accuracy and robustness. By combining multiple classifiers, stacking can mitigate the impact of biases inherent in individual models and improve the generalization capability of the overall predictive system.

Additionally, principal component analysis (PCA) is a powerful dimensionality reduction technique which is used for transforming complex datasets into a lower-dimensional space while retaining most of the essential information, PCA aids in simplifying data representations by linearly transforming the original features into orthogonal features known as principal components, which are ordered based on the variance they explain. Through the identification of eigenvectors and eigenvalues from the covariance matrix of the data, PCA captures the directions of maximum variance and their respective magnitudes [4–6]. PCA finds applications in various domains, including data visualization, noise reduction, and feature extraction [7, 8]. Through a pioneering method for predictive analysis in ischemic brain stroke utilizing advanced machine learning techniques i.e, diverse ML algorithms and ensemble learning strategies, proposed research has achieved exceptional predictive accuracy, reaching an impressive 98.6%.

Ensemble learning has become a focal point in the machine learning and computational intelligence fields because it offers a way to enhance prediction accuracy by pooling together multiple classifiers. While initially used to improve classification accuracy, ensemble methods have evolved to tackle a wide range of real-world issues such as adapting to changing concepts, correcting errors, selecting the most relevant features, learning incrementally, and estimating confidence levels. Researchers have delved deeply into various fusion techniques and the components that make up ensembles, leading to significant advancements in recent years [9–12].

The benefits of this research are multifaceted: enhanced prediction accuracy by combining multiple machine learning algorithms, efficient data utilization through proper data preprocessing and dimensionality reduction, early detection of high-risk individuals for timely intervention, support for personalized medicine by tailoring treatment plans, elucidation of key risk factors driving further research. Clinically, this method enables early detection of high-risk individuals, allowing for timely intervention and better resource allocation, and supports personalized medicine by tailoring treatment plans to individual risk profiles. Additionally, the approach aids research by elucidating key risk factors, driving further investigations into stroke prevention and treatment. Overall, this comprehensive method significantly contributes to early detection and prevention efforts, improving patient outcomes and addressing stroke-related healthcare challenges [13, 14].

This paper seeks to bridge the gap between machine learning and brain stroke identification. By harnessing the power of ensemble methods and classifier fusion, it aims to not only improve predictive accuracy but also streamline the process of identifying strokes early on. If successful, these advancements could revolutionize medical practices, paving the way for more effective interventions and ultimately saving lives.

### Motivation

We propose a pioneering approach to stroke prediction, leveraging advanced machine learning techniques and introducing a novel stacking methodology. Our research stands out for its innovative contribution in showcasing the robust performance of this stacking technique across a spectrum of crucial healthcare metrics. We demonstrate the potential of our proposed approach, thereby enhancing patient outcomes and healthcare management strategies.

### Literature survey

Stroke prediction research has witnessed significant advancements through the application of machine learning (ML) techniques, contributing to improved accuracy and timely interventions. This review synthesizes findings from recent studies focusing on ML approaches for stroke prediction, emphasizing algorithmic performance, feature selection methodologies, model interpretability, and key results.

In [15], an innovative stroke detection algorithm is presented, employing various ML classifiers such as Naïve Bayes, logistic regression, XgBoost, and support vector machines (SVM). Notably, the support vector machine algorithm outperformed other models, achieving exceptional accuracy (98.6%) and precision (99.9%). However, the paper lacks explicit discussions on feature selection and data preprocessing strategies.

In [16], researchers develop an ML-based stroke prediction algorithm utilizing readily available data from patients’ hospital presentations and investigating the impact of social determinants of health (SDoH) variables. The study reports high sensitivity and reasonable specificity of the ML stroke prediction algorithm, with significant improvements observed upon the inclusion of individual-level SDoH features. Importantly, experimental results demonstrate consistent outperformance of ML classifiers over logistic regression, with AUC improvements from 0.694 to 0.823 with the inclusion of SDoH features.

Moreover, [17] employs logistic regression (LR) with recursive feature selection (RFE) to predict stroke and Transient Ischemic Attack (TIA) diagnosis, highlighting the predictive utility of patient-reported symptoms. ML techniques achieve impressive performance metrics, with AUC exceeding 0.94 for stroke outcome prediction and notable enhancements upon incorporating follow-up data.

In [18], the stacking classification method emerges as a superior approach, showcasing high performance across multiple metrics, including an impressive AUC of 98.9% and an accuracy of 98%. The study underscores the efficacy of the stacking ensemble method, comprising base classifiers such as naive Bayes and random forests, with a logistic regression meta-classifier.

Additionally, [19] explores the interpretability of ML models for stroke prediction using SHAP and LIME techniques. Notably, Random Forest emerges as the top-performing algorithm with an accuracy score of 90.36%, followed closely by the XGB Classifier with an accuracy score of 89.02% [20–22].

In [23], machine learning (ML) is applied to predict early signs of ischemic stroke in emergency settings, although its predictive accuracy is constrained by the area under the receiver operating characteristic (AUC). The study highlights the XGBoost-based model’s superior predictive power for pre-screening ischemic stroke, particularly emphasizing the effectiveness of ML-based models using clinical laboratory features. Results showcase the XGBoost-based model’s highest accuracy in predicting ischemic stroke, alongside robust validation across multiple datasets. Additionally, the study demonstrates the XGBoost-based model’s ability to achieve high average sensitivities and specificities across training, internal validation, and external validation datasets, indicating its reliability for screening patients with ischemic stroke.

In [24], deep learning models are employed to forecast major adverse cerebrovascular events following acute ischemic stroke, furnishing personalized outcome predictions at an individual level. By leveraging clinical data and brain imaging, these models exhibit enhanced predictive accuracy for major adverse cerebrovascular events (MACEs) after acute ischemic stroke (AIS). Notably, deep learning techniques like DeepSurv and Deep-Survival-Machines surpass traditional survival models, marking a significant advancement in stroke prediction methodologies. Furthermore, the study provides comprehensive validation results, including AUC values and performance metrics such as sensitivity, specificity, classification accuracy, precision score, F1 score, and log loss across training, internal validation, and external validation datasets. These results underscore the reliability and robustness of deep learning models in predicting outcomes for AIS patients, thereby offering valuable insights for clinical decision-making and patient management [21, 25–27].

The reviewed literature also shown in Table 1 highlights the diverse ML approaches utilized in stroke prediction and their substantial results. These findings underscore the potential of ML techniques to enhance stroke risk assessment, thereby facilitating proactive interventions and improving patient outcomes. However, further research is warranted to address challenges related to feature selection, model interpretability, and real-world validation.

**Table 1:** Summary of machine learning approaches for stroke prediction

Study	Models used	Accuracy score	Importance
[15]	Naïve Bayes, Logistic Regression, XgBoost, SVM	SVM: 98.6%	SVM achieved the highest accuracy and precision (99.9%), highlighting its robustness.
[16]	Various ML classifiers, Logistic Regression	AUC: 0.694 to 0.823	Inclusion of SDoH features significantly improved AUC, showing the importance of these variables.
[17]	Logistic Regression with RFE	AUC:>0.94	Recursive feature selection and follow-up data incorporation enhanced predictive utility.
[18]	Stacking (Naïve Bayes, Random Forests, LR)	AUC: 98.9%, Accuracy: 98%	Stacking method demonstrated superior performance across multiple metrics.
[19]	Random Forest, XGBoost	Random Forest: 90.36%, XGBoost: 89.02%	SHAP and LIME techniques enhanced interpretability, with Random Forest performing best.
[23]	XGBoost	Highest Accuracy	XGBoost showed superior predictive power for pre-screening ischemic stroke.
[24]	DeepSurv, Deep-Survival-Machines	Enhanced Predictive Accuracy	Deep learning models surpassed traditional survival models for predicting MACEs after AIS.

### Aim

This research aims to pioneer a pioneering approach to predictive analysis of Ischemic brain stroke with machine learning techniques. Initially, the study focuses on utilizing preference algorithms to discern the key traits using several machine learning techniques such as Logistic regression, support vector machine, decision tree and K-nearest neighbor. We utilized PCA for the reduction the dimensionality of the dataset.

Contributions of our study as follows:

- Demonstrated the effectiveness of Principal Component Analysis (PCA) in optimizing model accuracy for stroke prediction.
- Identified an optimal PCA configuration, specifically with 16 components, achieving a significant improvement in predictive performance.
- Implemented a stacking ensemble method combining Random Forest, Decision Tree, and K-Nearest Neighbors (KNN), resulting in a high accuracy of 98.6%.
- Showcased the potential of advanced machine learning techniques in enhancing stroke risk assessment and guiding preventive healthcare strategies.

The subsequent sections of this paper are organized as follows: in Sect. 2, we elaborate on the feature Selection method and Classifier. Following that, in Sect. 3, we present the experiment and results of our study, including a comparative analysis of our model with both the proposed model and other state-of-the-art methods.

## Methodology

### Dataset

This dataset from Kaggle includes 5110 patients, with attributes such as gender, age, presence of hypertension, history of heart disease, marital status, type of work, residence type, average glucose level, body mass index (BMI), smoking status, and stroke occurrence. The gender attribute is categorical, the age is numerical, and hypertension and heart disease are binary indicators (1 for yes, 0 for no). Marital status is recorded as either married or not married, while work type categories include government job, never worked, private, self-employed, and children. Residence type is categorized as urban or rural. Average glucose level and BMI are continuous variables, and smoking status is categorized as never smoked, formerly smoked, or smokes. The target variable is stroke prediction, also a binary indicator (1 for stroke, 0 for no stroke). For every column, there are comprehensive explanations in Table 2.

**Fig. 1:**
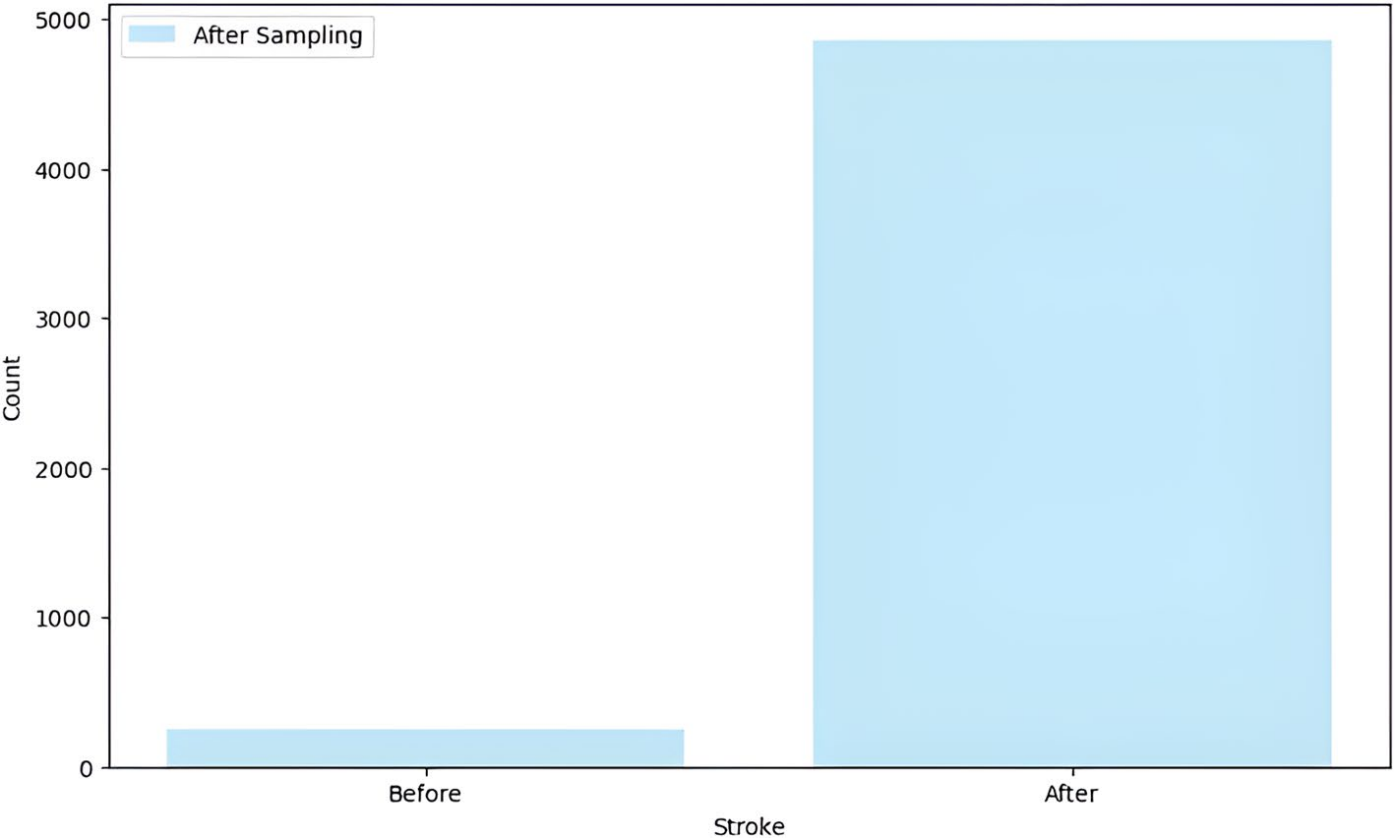
Distribution of stroke and no stroke cases before and after oversampling

To rectify dataset imbalances and bolster model accuracy, we implement oversampling techniques. We aim to equalize representation across classes by increasing the number of instances in the minority class (stroke) to match that of the majority class (no stroke). Post-oversampling, both classes comprise 4861 cases each, ensuring a balanced dataset for training and testing. The disparity in stroke class distribution pre- and post-oversampling is visually depicted in the accompanying image. Figure 1 depicts for the same.

We use the following features from the stroke prediction dataset, which is publicly available on Kaggle. Table 2 provides a detailed description of each feature.

**Table 2:** Dataset features summary: feature, description, data type, and additional information

Feature	Description	Data type	Additional information
Gender	Patient’s gender	Object	
Age	Patient’s age	Float64	
Hypertension	Presence of hypertension	Int64	1: yes, 0: no
Heart_disease	History of heart disease	Int64	1: yes, 0: no
Ever_married	Marital status	Object	Married/not married
Work_type	Type of work	Object	Govt_job/Never_worked/Private/ Self-employed/children
Residence_type	Residence type	Object	Urban/rural
Avg_glucose_level	Average glucose level	Float64	
BMI	Body mass index	Float64	
Smoking_status	Smoking status	Object	Never smoked/formerly smoked/smokes
Stroke	Stroke prediction	Int64	1: stroke found, 0: stroke not found

**Fig. 2:**
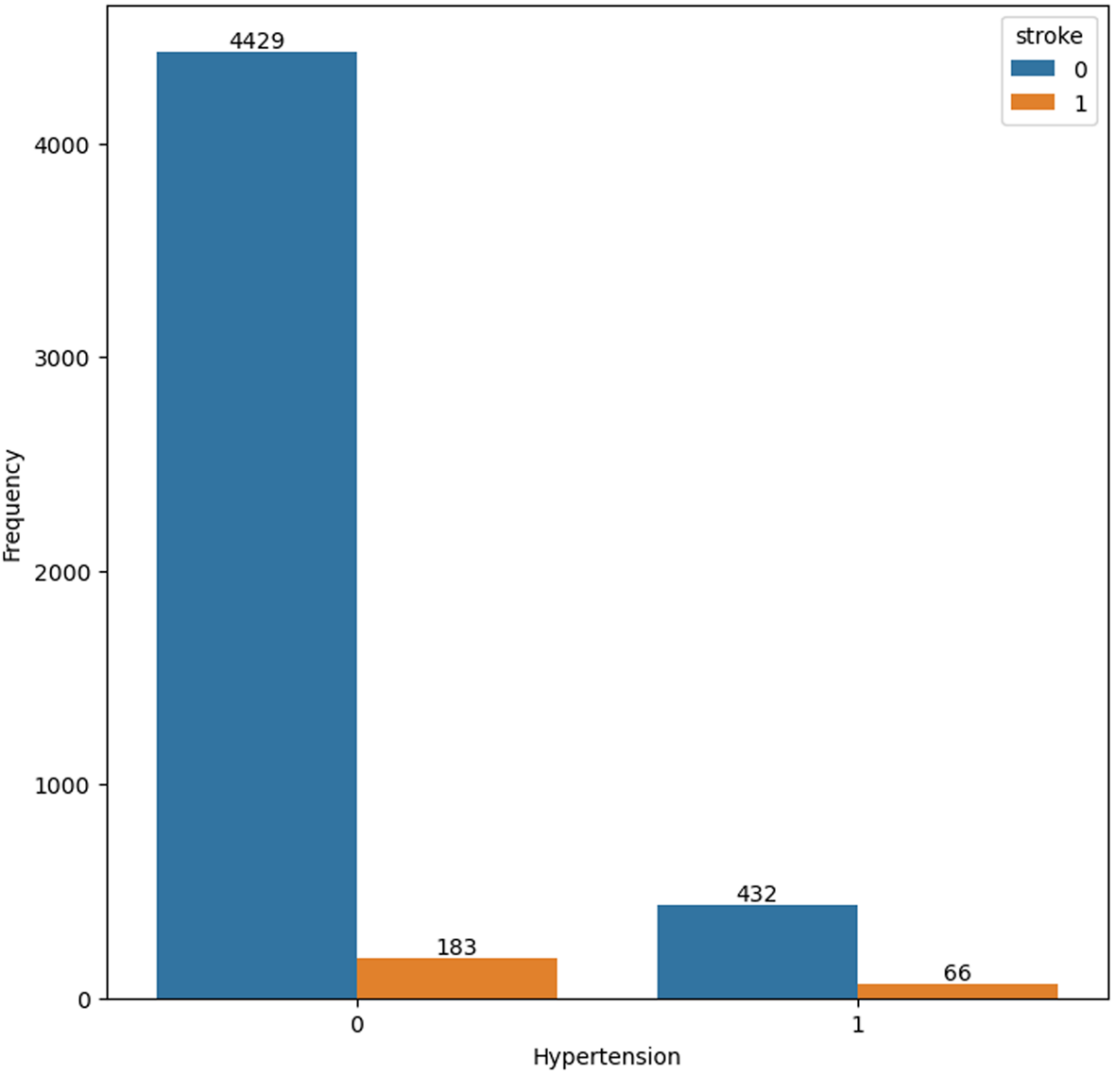
Count-plot for hypertension cases in the dataset

**Fig. 3:**
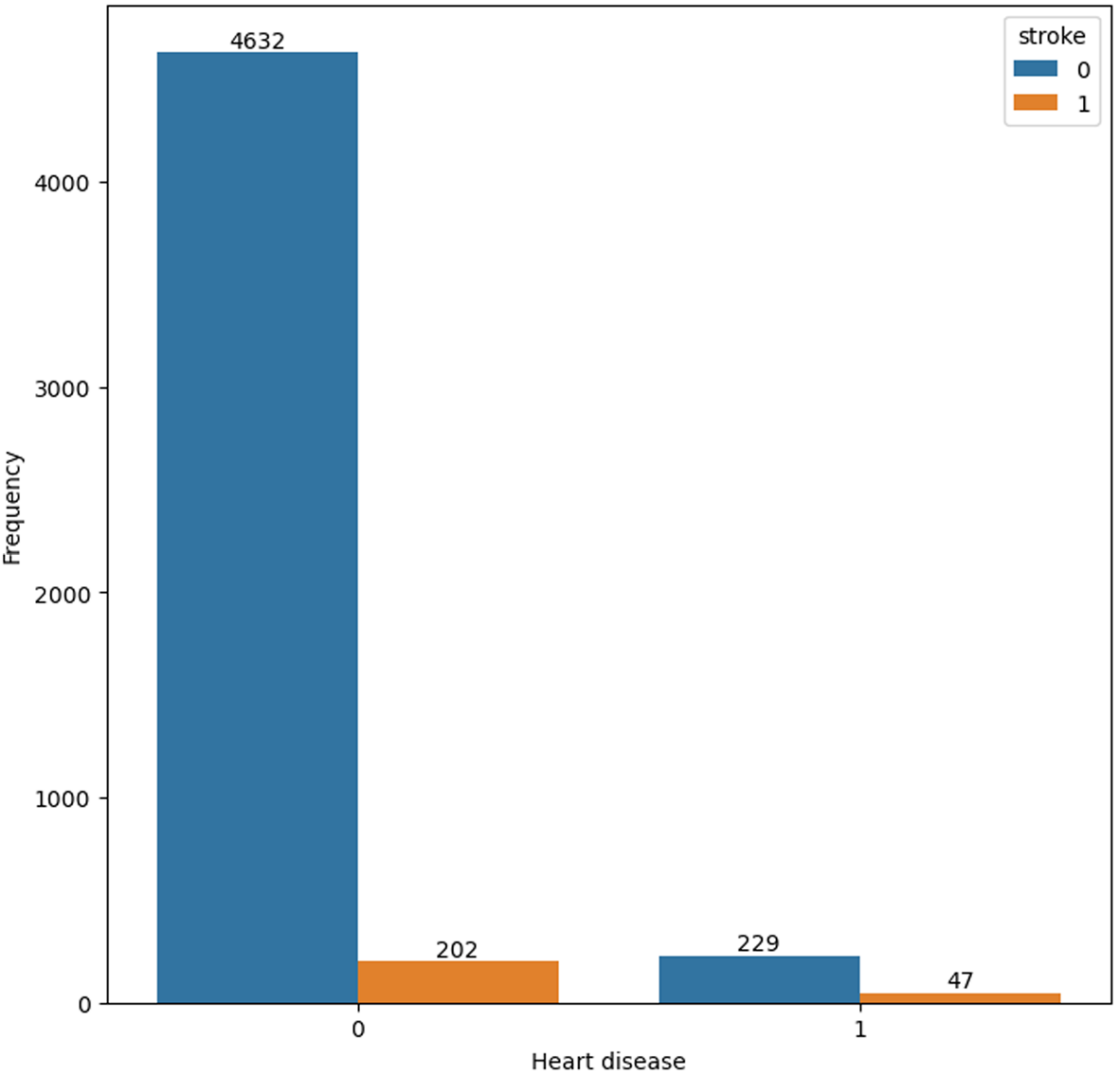
Count-plot for heart disease cases in the dataset

Figures 2 and 3 depict the prevalence of heart disease and hypertension among participants who have experienced a stroke. In both figures, a significant proportion of participants who have had a stroke do not have a diagnosis of hypertension or heart disease.

**Fig. 4:**
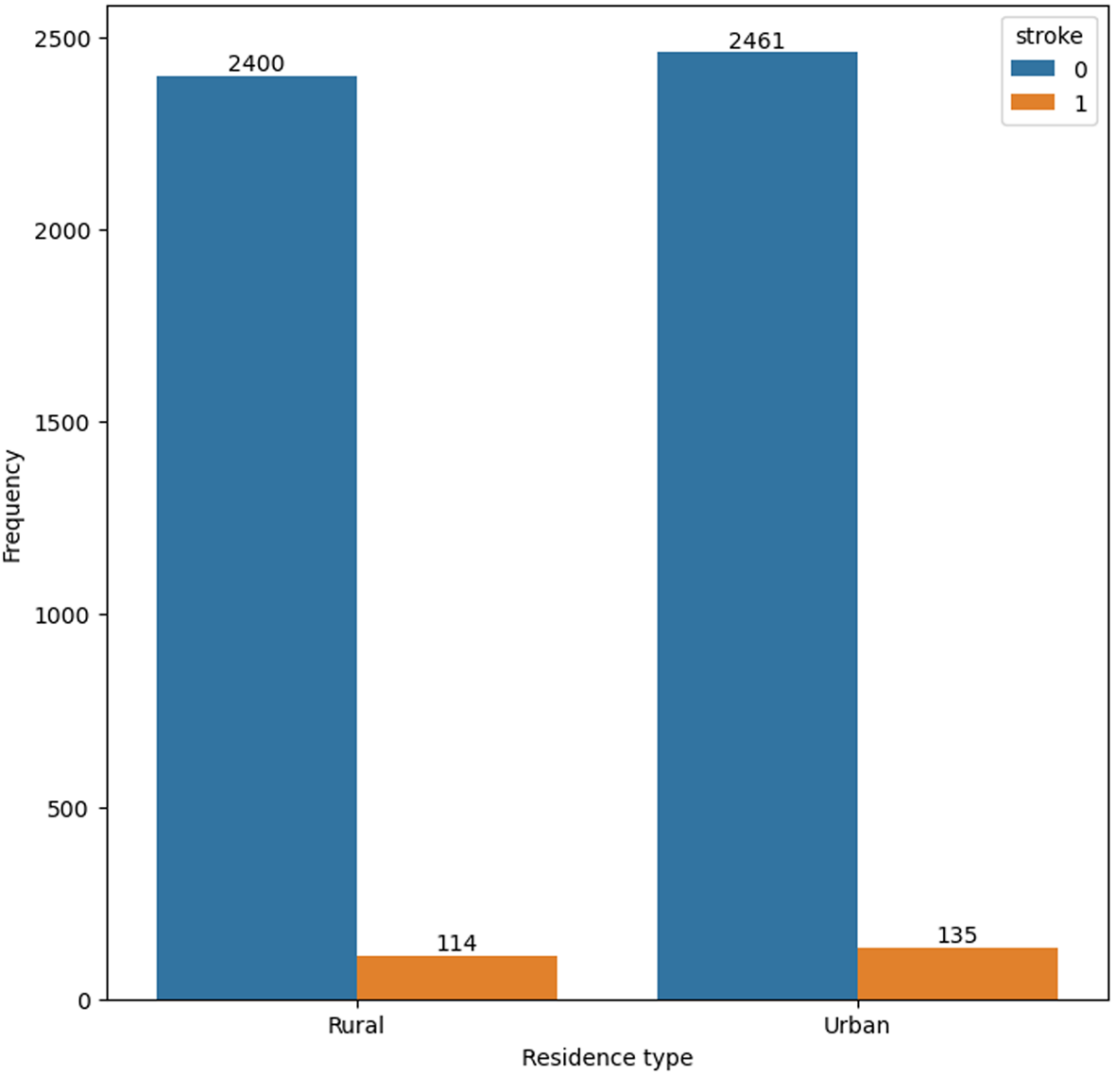
Distribution of resident types in the dataset

**Fig. 5:**
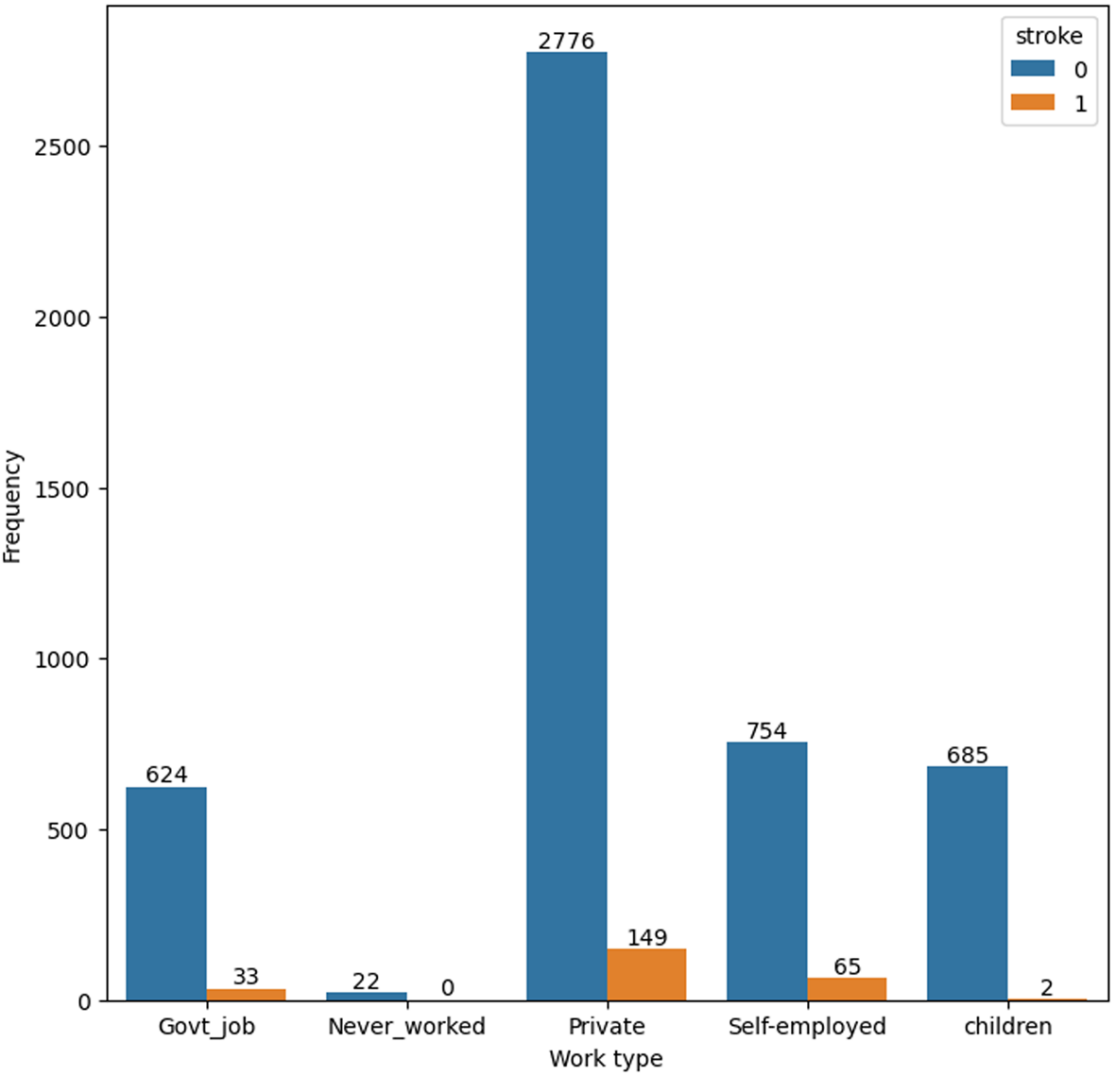
Distribution of work type in the dataset

Figures 4 and 5 display the prevalence of residence type and work type among participants who have experienced a stroke. These figures highlight that a significant proportion of participants who have had a stroke reside in urban areas and have a private work type.

**Fig. 6:**
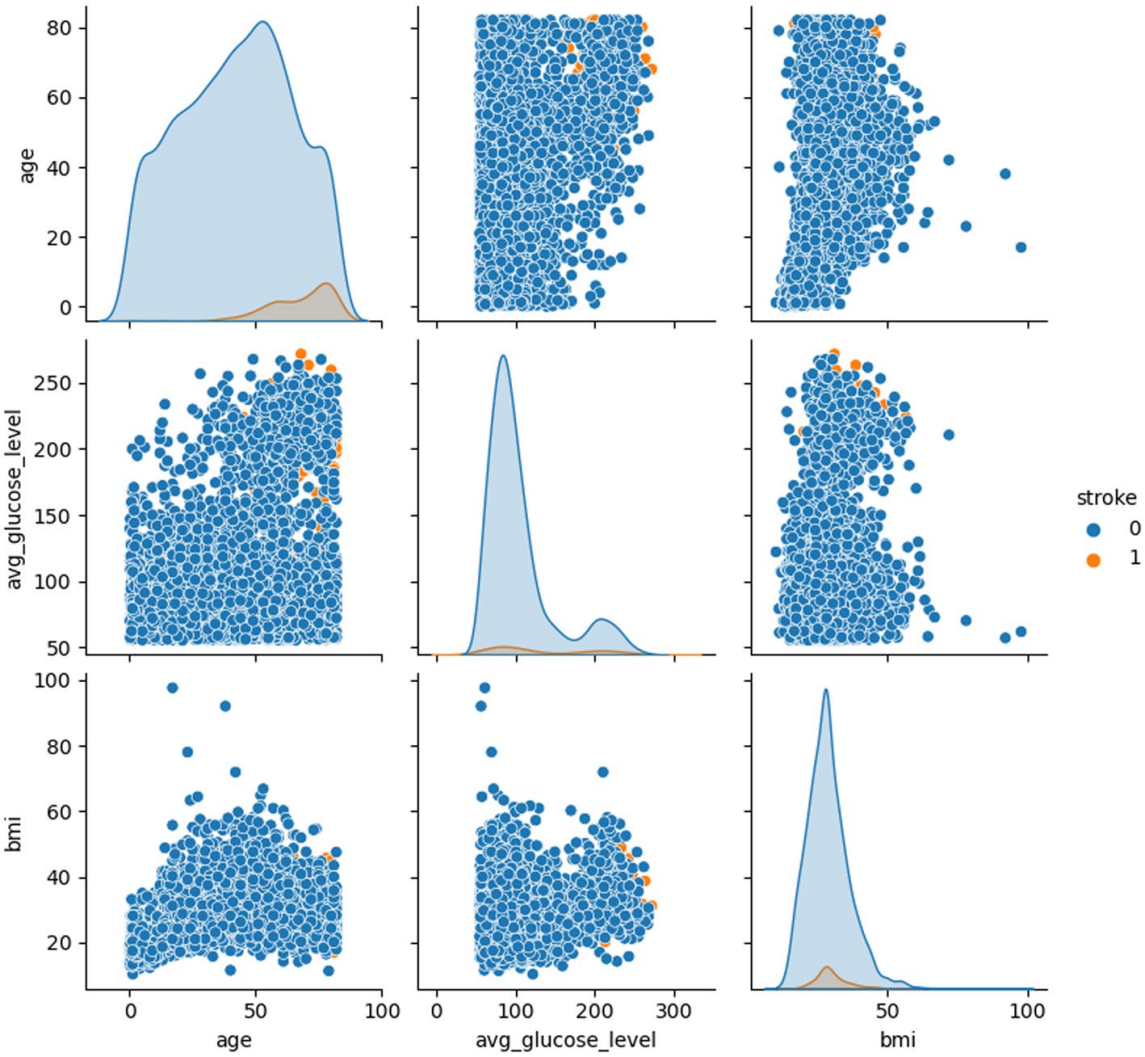
Distribution of glucose in the dataset

**Fig. 7:**
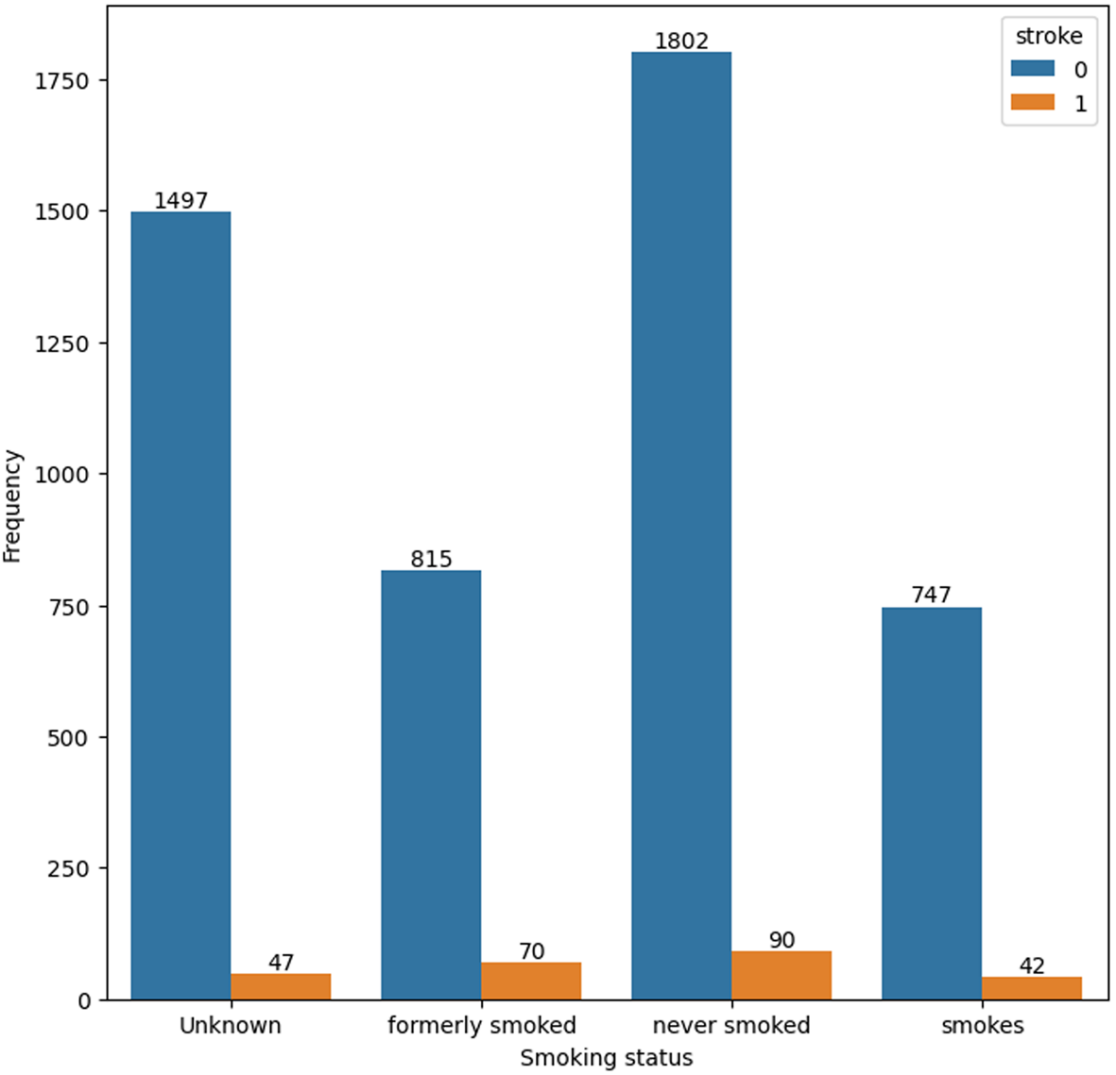
Distribution of smoking status in the dataset

Figures 6 and 7 display the prevalence of glucose level and smoking level among participants who have experienced a stroke.

**Fig. 8:**
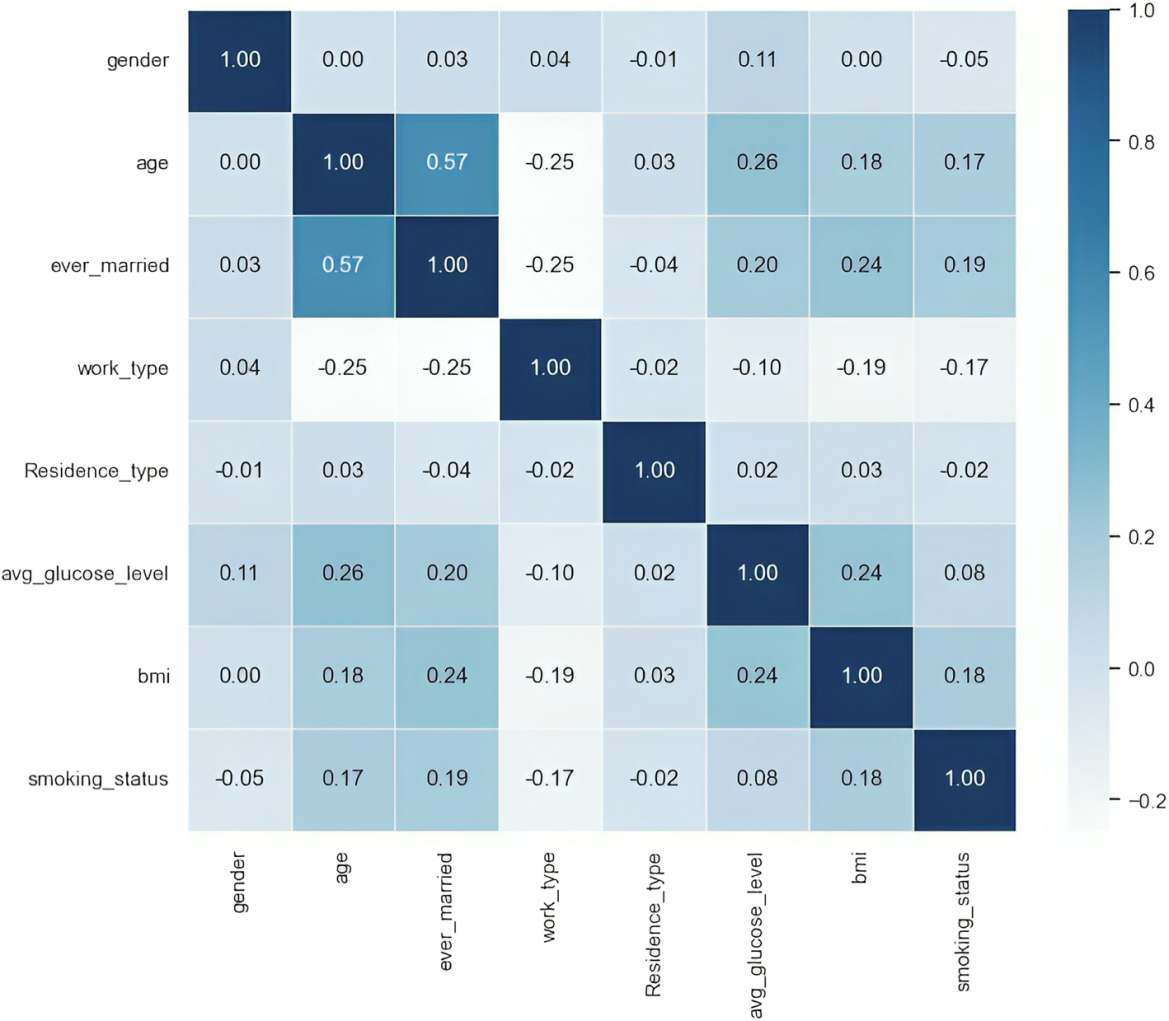
Correlation matrix of variables in the stroke dataset

Figure 8 display the correlation among various features. The figure provides valuable insights into the interplay and potential dependencies among these attributes, which are crucial for understanding the underlying patterns and dynamics within the dataset.

### Data pre-processing

Ensuring the quality of raw data is crucial for the accuracy of our final predictions, particularly in the presence of missing values and noisy data. Therefore, our research emphasizes the necessity of data preprocessing to enhance the appropriateness of the data for analysis. This preprocessing involves several steps, including the reduction of redundant values, feature selection, and data discretization.

An integral part of our data preprocessing strategy is addressing class imbalance, a common challenge in predictive modeling. To tackle this issue, we employ the Synthetic Minority Over-sampling Technique (SMOTE) within our proposed framework. By oversampling the minority class, specifically the ’stroke’ participants, we aim to achieve a more balanced distribution, thereby preventing biases in the predictive model.We addressed missing values within the BMI column by imputing them with the median value. This method ensures that the dataset remains robust and complete for subsequent analysis.

**Fig. 9:**
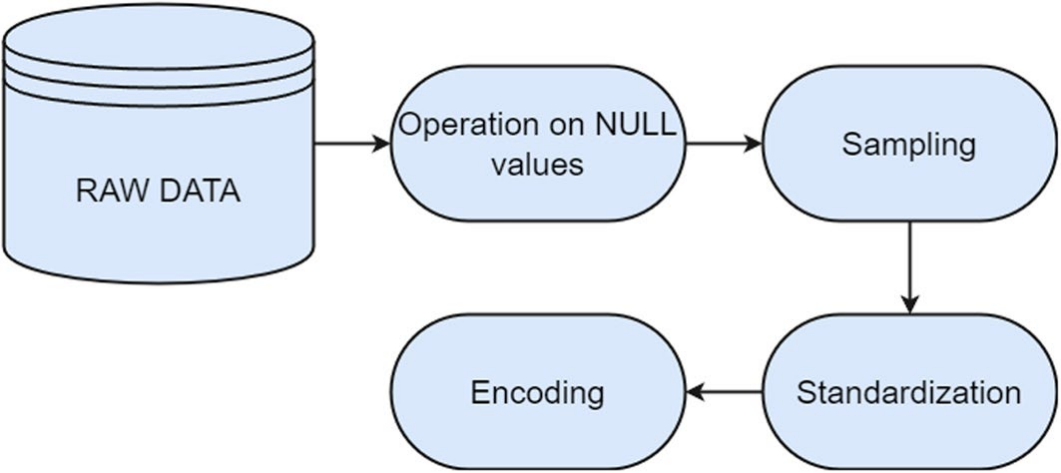
Flowchart illustrating data preprocessing steps

Figure 9 shows us the end-to-end flow charts of the preprocessing.

**Algorithm 1:**
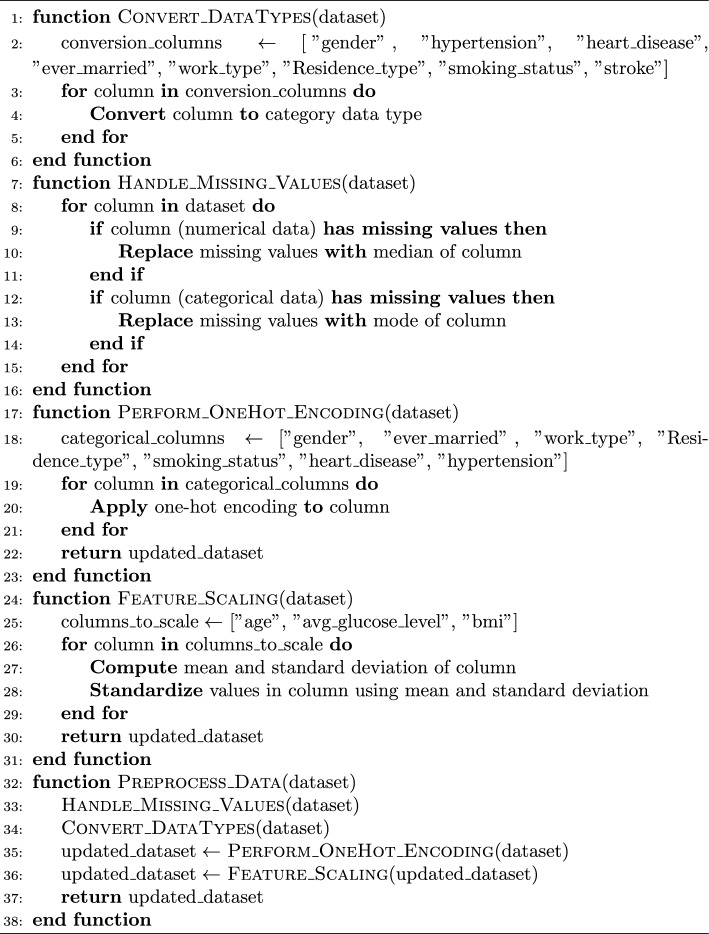
Data preprocessing

### Feature selection

This study explores the impact of varying the number of components in Principal Component Analysis (PCA) on the accuracy of stroke prediction models. By systematically adjusting the value of n from 1 to 16, we observed that the majority of models exhibited the highest accuracy when n was set to 16. Building upon this observation, we proceeded to implement a stacking ensemble method. In this approach, we combined the predictions from the three best-performing models: Random Forest and Decision Tree as base estimators, and K-Nearest Neighbors (KNN) as the final estimator.

Upon applying the stacking ensemble technique, we achieved a remarkable accuracy of 98.6%. This significant improvement underscores the efficacy of combining complementary strengths from multiple models to enhance predictive performance.

This research aims to compare the performance of various machine learning classifiers in predicting stroke occurrences after dimensionality reduction using PCA. We utilized PCA to reduce the dimensionality of the dataset and then trained several classifiers including Random Forest, SVM, XGBoost, Naive Bayes, KNN, Logistic Regression, and Decision Tree on the transformed data.

Before training the models, we conducted data preprocessing steps including handling missing values (replacing them with the median value for BMI), feature scaling, and splitting the data into training and testing sets. Each classifier was evaluated using accuracy scores, F1 scores, precision, and recall which were computed by comparing the model predictions with the actual labels in the test set.

The results of our analysis are presented in a data frame, showcasing the accuracy of each classifier for different numbers of PCA components. Some key risk factors can be identfied as:

- *Age:* Older age significantly increases the risk of ischemic stroke.
- *Hypertension:* High blood pressure is a major risk factor.
- *Diabetes:* Diabetes mellitus is strongly associated with an increased risk.
- *Smoking:* Tobacco use contributes to the risk of stroke.
- *Cholesterol levels:* High levels of LDL cholesterol can lead to stroke.
- *Cardiovascular diseases:* Conditions like atrial fibrillation and heart failure are critical predictors.
- *Lifestyle factors:* Physical inactivity, poor diet, and obesity are important considerations.
- *Genetic factors:* Family history and specific genetic markers can also be significant.

These factors are typically integrated into machine learning models to enhance the prediction accuracy of ischemic stroke outcomes.

The findings demonstrate that the performance of the classifiers varies with the number of PCA components, with certain classifiers exhibiting better accuracy than others. This information can guide the selection of an appropriate classifier for stroke prediction tasks based on the desired trade-off between computational complexity and predictive accuracy.

**Algorithm 2:**
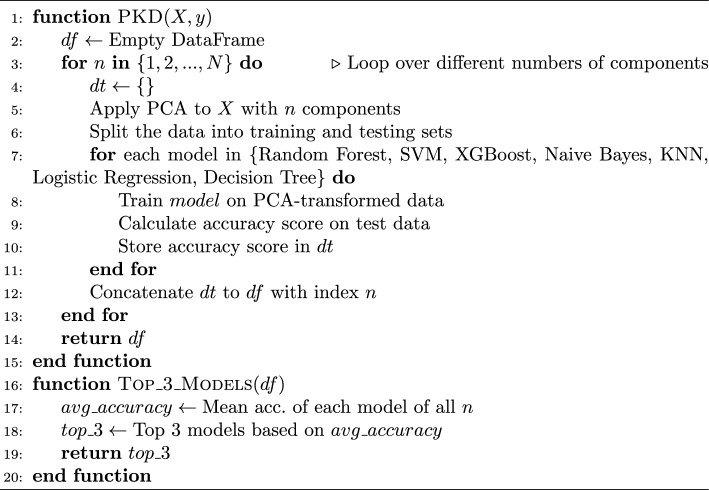
Feature selection

### Classification

In our research paper, we’ve employed cutting-edge classification techniques to predict and mitigate the risk of stroke occurrences. Stacking, a sophisticated ensemble learning method, has been at the forefront of our approach, allowing us to amalgamate insights from base classifiers. This innovative fusion of classifiers has enabled us to discern intricate patterns and relationships in patient data, enhancing the precision and reliability of our predictive models.

Our methodology involved training a diverse ensemble of classifiers on comprehensive dataset. These classifiers, acting as the foundation, have collectively contributed to our understanding of stroke risk factors and prediction accuracy. Through iterative refinement and model aggregation facilitated by stacking, we’ve strived to push the boundaries of stroke prediction, aiming for more personalized healthcare interventions and improved patient outcomes.

#### Technical details

*Principal component analysis (PCA):* PCA was employed for dimensionality reduction, standardizing data, computing the covariance matrix, and projecting data onto principal components to retain 95% variance.

PCA assumes linearity and Gaussian distributions in the data, which may not always be applicable. This powerful dimensionality reduction technique some specific features in stroke prediction which provides valuable insights to medical professionals. In this context they are listed below:

1. *Variance capture:* PCA identifies and retains the components that explain the highest variance in the data, ensuring that the most informative aspects are prioritized.
2. *Noise reduction:* By filtering out less significant components, PCA reduces noise, which helps in making the prediction model more robust and accurate.
3. *Multicollinearity handling:* PCA transforms correlated features into uncorrelated principal components, addressing issues of multicollinearity that can affect model performance.
4. *Simplification:* It reduces the complexity of the dataset by lowering the number of features, which simplifies the model and enhances computational efficiency.

*Base classifiers configuration:*

- *Random forest:* 500 trees, criterion = ‘entropy’, max depth = None, min samples leaf = 1, min samples split = 5
- *Decision tree:* criterion = ‘entropy’, max depth = None, min samples leaf = 1, min samples split = 5

*Stacking classifier training:*

- *Base classifier training:* Each base classifier was independently trained on the training dataset.
- *Level 1 data generation:* Predictions from base classifiers were used to generate a new dataset, serving as input for the meta-classifier. This involved performing 5-fold cross-validation on the training set to avoid overfitting.
- *Meta-classifier (final estimator):* K-nearest neighbors (KNN) with 5 neighbors and Euclidean distance metric.

*Training and evaluation:* The dataset was split into 80% training and 20% validation sets. Fivefold cross-validation was performed to tune hyperparameters and evaluate each classifier’s performance. Metrics such as accuracy, precision, recall, and F1-score were used to assess the stacking classifier’s effectiveness.

This comprehensive and detailed approach ensures robust and accurate stroke risk predictions, paving the way for personalized healthcare interventions and improved patient outcomes.

## Experiment and results

### Experimental setup

To replicate our experiments, the following hardware and software were used:

*Hardware*

CPU: Intel Core i7-9700K @ 3.60GHz GPU: NVIDIA GeForce RTX 2080 RAM: 32GB DDR4 Storage: 1TB SSD

*Software*

Operating system: Ubuntu 20.04 LTS Programming Language: Python 3.8 Libraries: Scikit-learn 0.24.2 for machine learning algorithms NumPy 1.20.3 for numerical computations Pandas 1.3.3 for data manipulation Matplotlib 3.4.3 and Seaborn 0.11.2 for data visualization TensorFlow 2.6.0 and Keras 2.6.0 for deep learning models Development Environment: Jupyter Notebook and PyCharm These specifications provide a baseline for replicating our study and further developing the predictive model for ischemic stroke.

### Evaluation metrics

In our investigation into predicting ischemic stroke occurrences, we evaluated the performance of our predictions by comparing them against actual data using predefined metrics. The dataset encompasses diverse patient characteristics pertinent to stroke prognosis.

Evaluation metrics are critical for analyzing the performance of categorization models. Accuracy is the proportion of properly identified cases overall, providing a broad measure of model performance. Precision highlights the fraction of true positive forecasts among all positive predictions, indicating how reliable positive predictions are. Recall, on the other hand, emphasizes the fraction of true positive predictions across all actual positive cases, demonstrating the model’s capacity to detect positives. Specificity is the proportion of genuine negative predictions among all real negative cases, demonstrating the model’s ability to identify negatives correctly. The F1-Score, which is the average of the harmonics of precision and recall, gives a balanced assessment that is especially beneficial in circumstances with uneven class distributions. These measurements provide insights into a model’s strengths and limitations, aiding in the Helping in maximizing efficiency and choosing the suitable models for classification jobs.

### Performance of proposed method

**Table 3:** Benchmarking various algorithmic approaches

Model	TP	FP	FN	TN	Acc. (%)	Pre. (%)	Rec. (%)	F1-score (%)
KNN	814	170	157	827	83.3	82.7	83.8	83.2
NN	726	248	207	787	76.8	74.5	77.8	76.1
RF	735	237	218	718	76.8	75.6	77.1	76.3
SVM-L	494	487	475	512	51.1	50.3	50.9	50.6
SVM-R	651	275	219	823	74.8	70.3	74.8	72.4
ADA	747	235	168	836	80.4	76.1	81.6	78.7
MNB	630	309	226	803	72.8	67.0	73.6	70.1
Proposed method	942	20	3	1004	98.6	97.9	99.6	98.7

TP, true posistive; FP, false positive; FN, false negative; TN, true negative; Acc, accuracy; Pre, precision; Rec, recall

**Table 4:** Accuracy scores with various proposed algorithms

Algorithms	Accuracy (%)
Hybrid machine learning approach	71.6
GBT	78
SQMLP	86.78
Proposed method	98.6

**Fig. 10:**
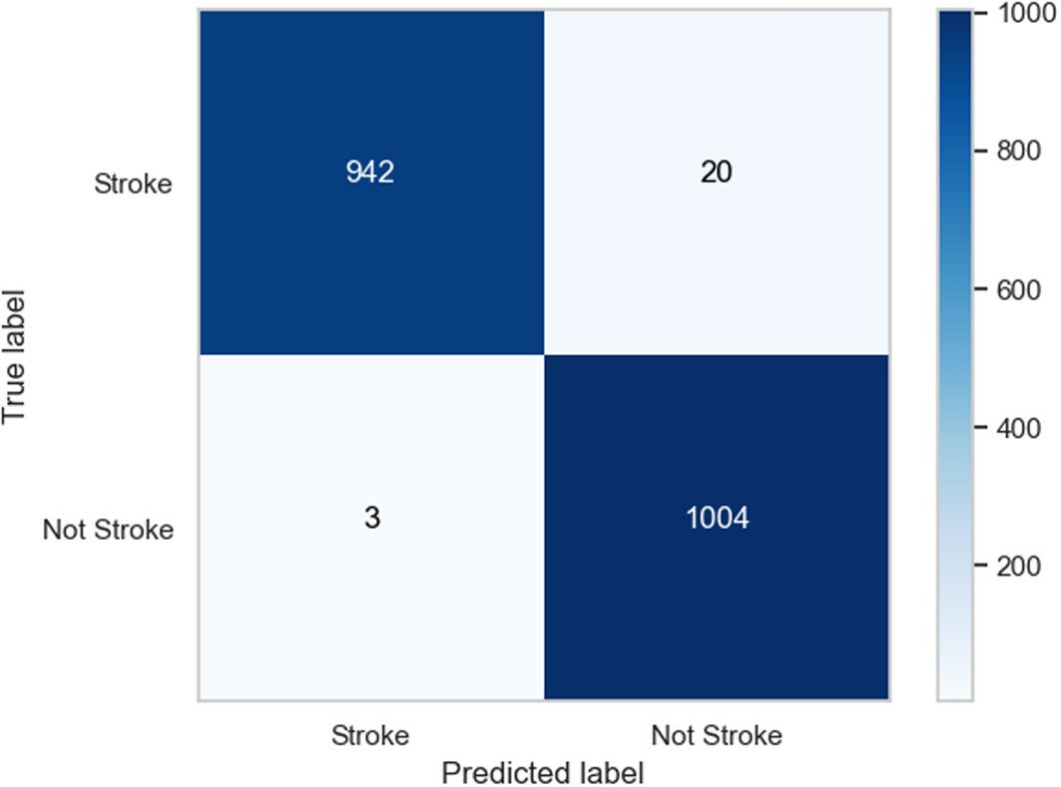
Confusion matrix of predicted versus actual classes of our proposed method

**Fig. 11:**
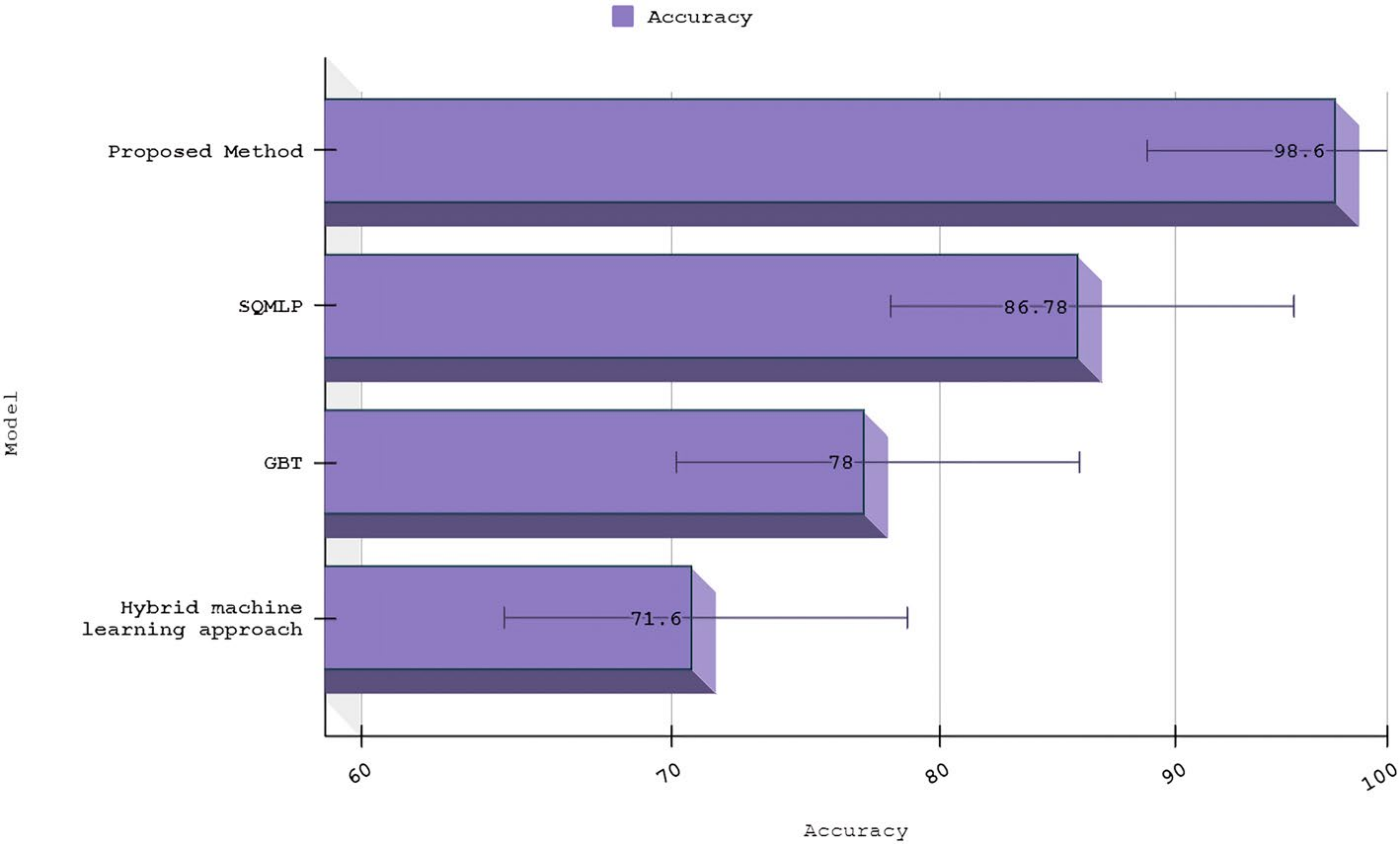
Comparative testing accuracy of different models

Our algorithm’s precision is compared to other machine learning methods And a specific comparison is present in Table 3; not only that, we have compared other proposed methods with our method, Fig. 11 demonstrates the contrast of various state-of-the-art models and data refer Table 4. We used the confusion matrix in Fig. 10 to obtain a better understanding of our model’s performance. We can pinpoint particular areas of strength or weakness in terms of accurately recognizing various classes or categories within the dataset by examining the matrix.

**Fig. 12:**
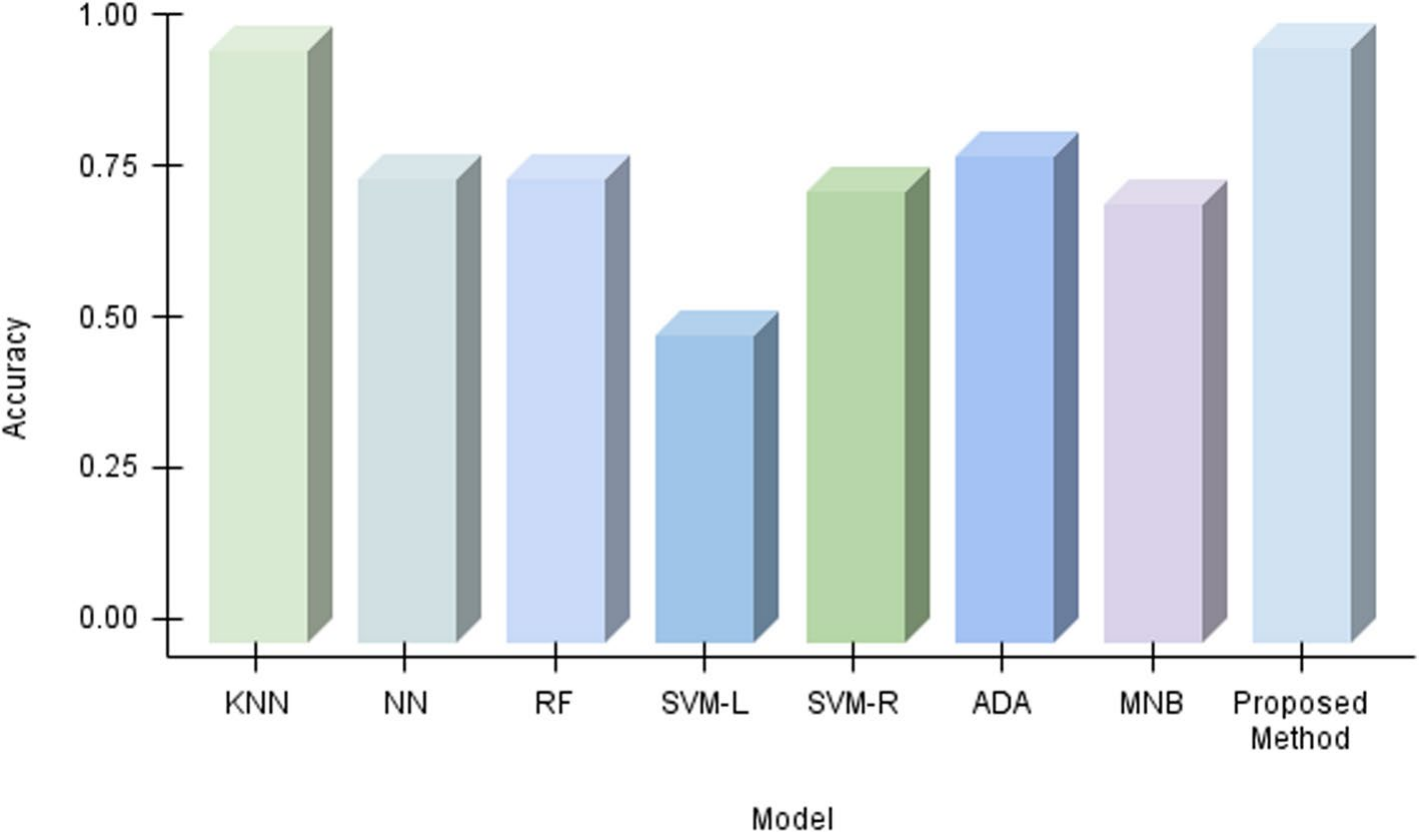
Testing accuracy of all machine learning models

**Fig. 13:**
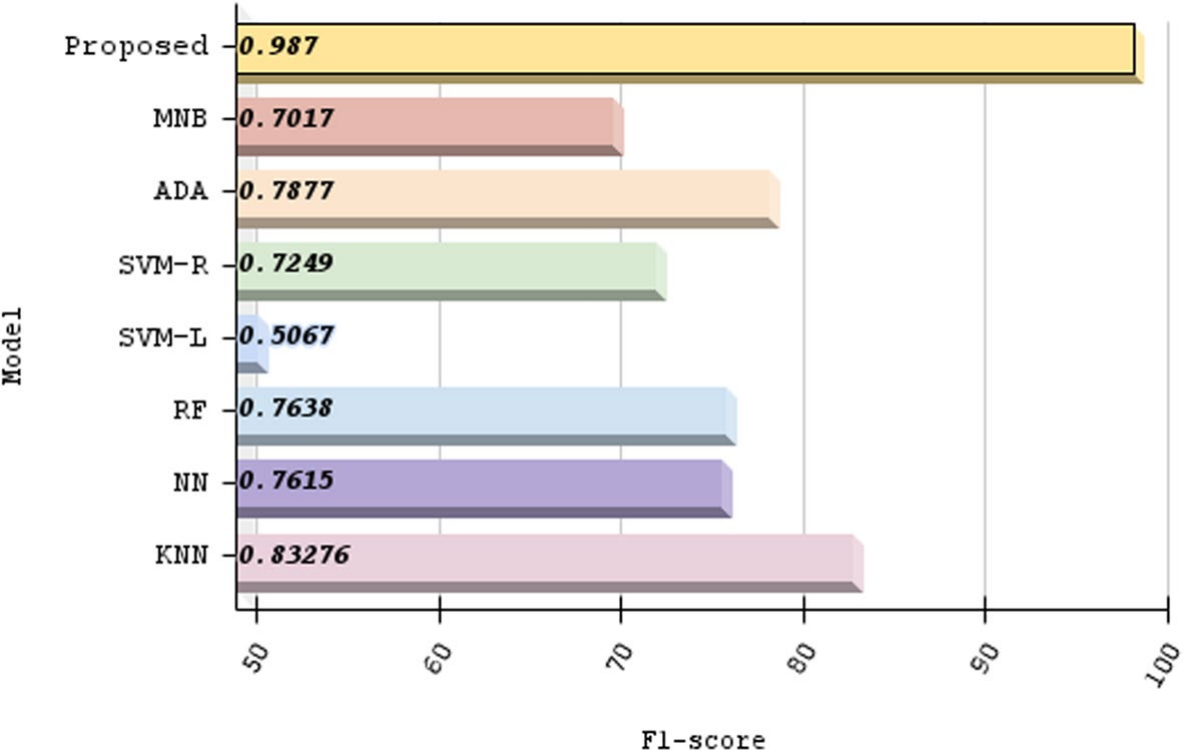
F1 score comparison with other machine learning models

**Fig. 14:**
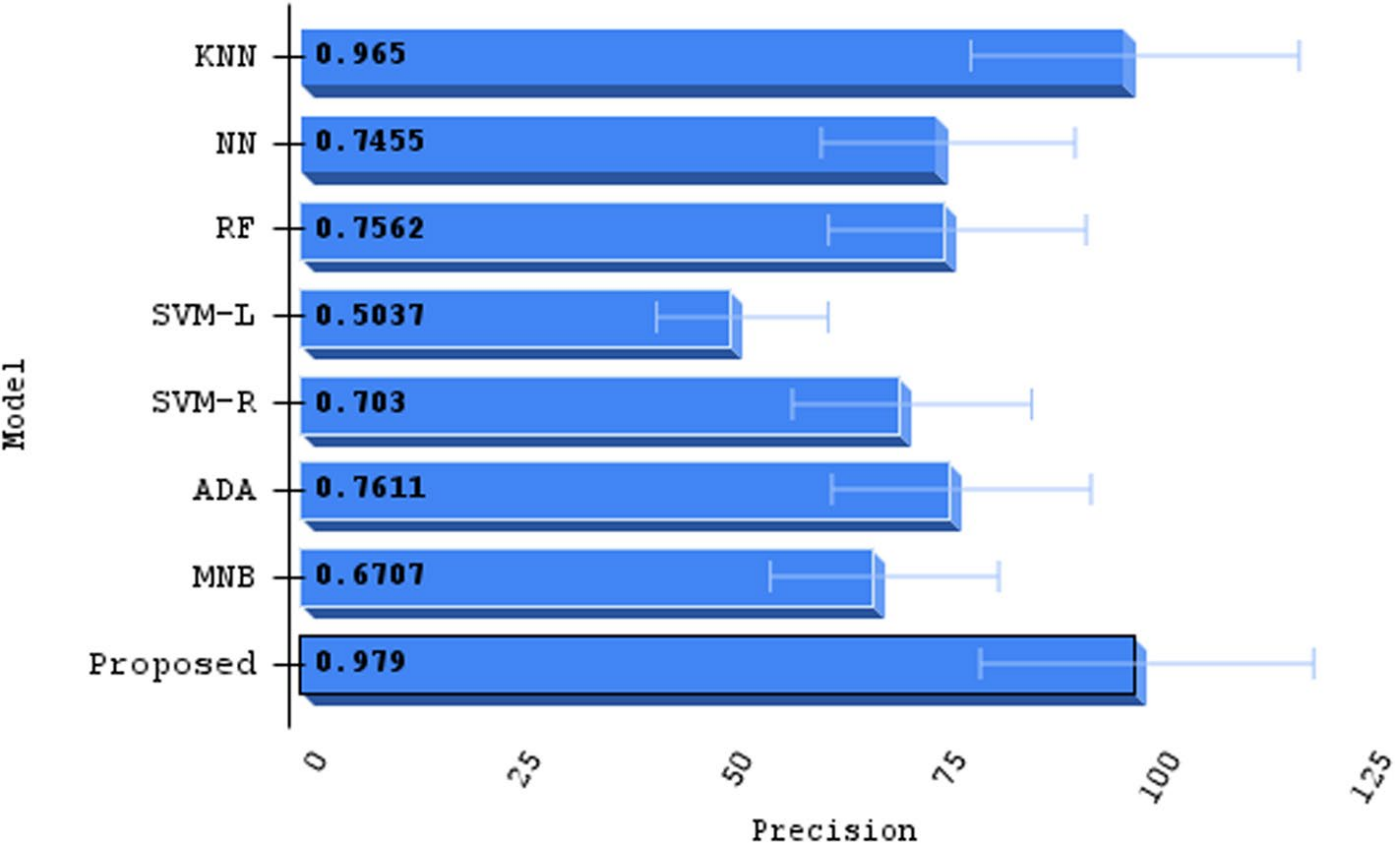
Precision comparison with other machine learning models

**Fig. 15:**
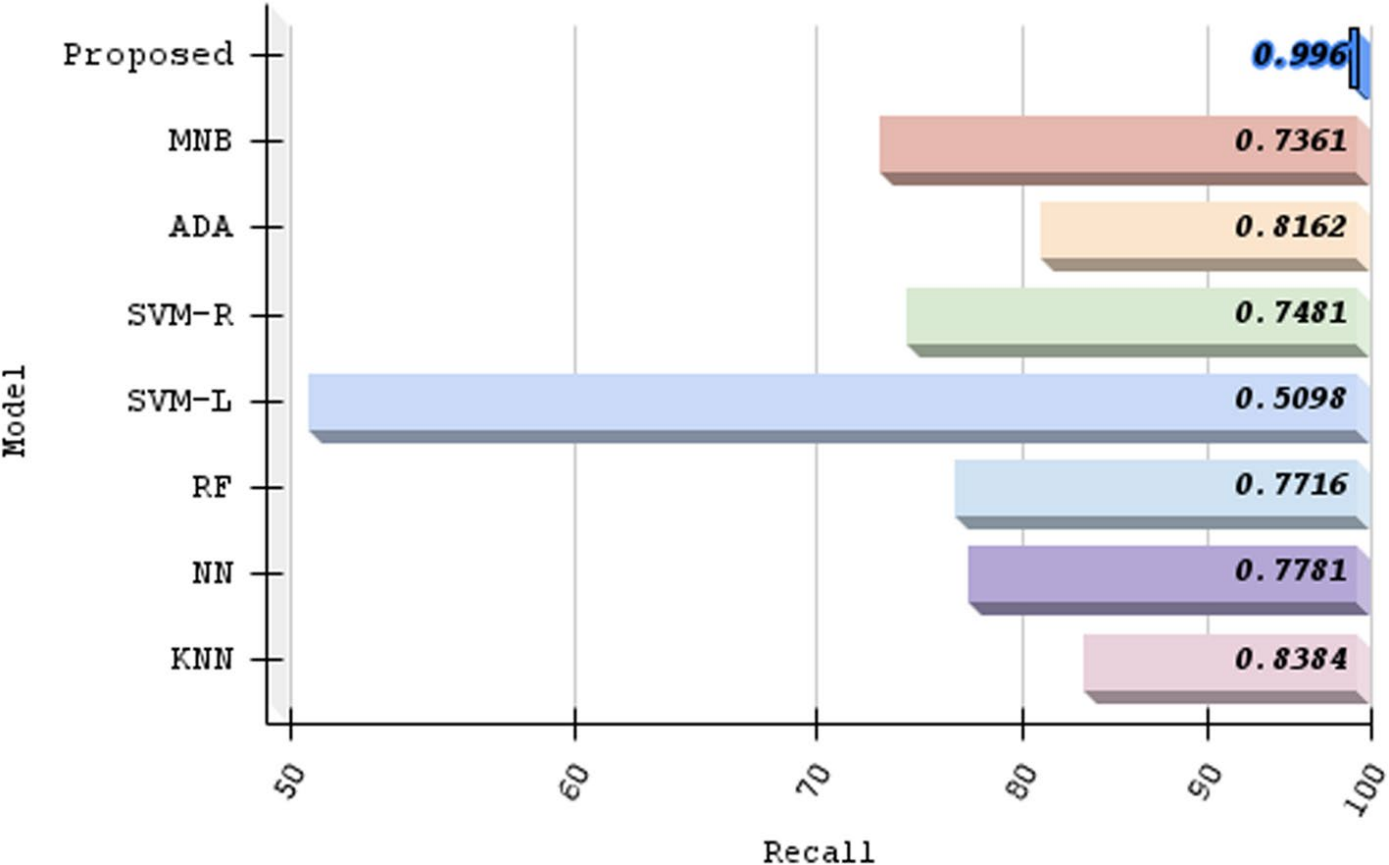
Recall comparison with other machine learning models

Our approach outperformed machine learning standards in predicting ischemic stroke, with an impressive accuracy of 98.6%. The evaluation, shown in Fig. 11 and described in Table 4, placed our proposed method as a forerunner in the field. Comparative assessments of other proposed methods demonstrated their superiority. SQMLP obtained 86.78% accuracy, while GBT yielded 78% accuracy. In contrast, a hybrid machine learning technique attained an accuracy of 71.6%. Our model’s higher predictive skills in comparison to existing models illustrate its efficacy in predicting ischemic strokes (Figs. 12, 13, 14, 15).

We verified our proposed method’s performance against established machine learning techniques (refer to Table 3). SVM-L had an accuracy of 51.118%, LR at 77.714%, MNB at 72.815%, SVM-R and NN at 74.88%, RF and KNN at 76.88%, and ADA at 80.437%. The method we used exceeded standards with an amazing accuracy of 98.6%. This significant achievement highlights the effectiveness of our approach, demonstrating its capacity as a successful instrument in stroke with ischemia prediction. Below we have provided a thorough analysis of the advantages and disadvantages of our proposed method:

- *Advantages*
  - *Enhanced sensitivity:* The stacking method helps in reducing false negatives by combining the strengths of multiple classifiers. This means that patients who are at risk of stroke but might be overlooked by a single model are more likely to be correctly identified.
  - *Robustness to variability:* By leveraging different algorithms, the model can better handle variability in patient data, which reduces the chance of missing true stroke cases.
  - *Improved generalization:* The ensemble approach improves the generalization capability of the method, thus enhancing its ability to correctly identify at-risk patients across different subgroups within the dataset.
- *Disadvantages*
  - *Complexity and interpretability:* The stacking method increases the complexity of the proposed method, making it more difficult to interpret. This can be a drawback when explaining decisions to medical professionals or patients.
  - *Resource intensive:* Training and tuning multiple base classifiers and a meta-classifier require more computational resources and time, which can be a limitation in resource-constrained environments.
  - *Potential overfitting:* Despite efforts to avoid overfitting through techniques like cross-validation, there is still a risk that the stacked method could overfit to the training data, potentially leading to missed stroke cases in unseen data.
  - *Generalizability:* The effectiveness of the proposed methods may vary across different datasets and population demographics. Further validation on diverse datasets is necessary to assess its applicability in various clinical settings.
  - *Data size limitation:* The study may be constrained by the size and diversity of the dataset used. Larger datasets with more comprehensive features could provide further insights and improve model robustness.

### TOPSIS analysis

The technique for order of preference by similarity to ideal solution (TOPSIS) is a method used for ranking and selection of alternatives based on their closeness to the ideal solution. The following subsections outline the steps involved in applying the TOPSIS method.

#### Normalize the decision matrix

We begin by normalizing the decision matrix. This step converts the various criteria dimensions into non-dimensional criteria, allowing comparisons across the different criteria. The normalization is done using the following formula:

1 $$\begin{aligned} r_{ij} = \frac{x_{ij}}{\sqrt{\sum _{i=1}^m x_{ij}^2}} \end{aligned}$$

where $r_{ij}$ is the normalized value, $x_{ij}$ is the original value, $i$ is the index of the alternative, and $j$ is the index of the criterion.

Refer to Table 5 for the normalized decision matrix.

**Table 5:** Normalized decision matrix

Model	Accuracy	Precision	Recall	F1-score
KNN	0.83396	0.82716	0.8384	0.83276
NN	0.7688	0.7455	0.7781	0.7615
RF	0.7688	0.7562	0.7716	0.7638
SVM-L	0.51118	0.5037	0.5098	0.5067
SVM-R	0.7488	0.703	0.7481	0.7249
ADA	0.80437	0.7611	0.8162	0.7877
MNB	0.72815	0.6707	0.7361	0.7017
Proposed method	0.986	0.979	0.996	0.987

#### Obtain the weighted standardized decision matrix

Since all criteria are considered equally important, each criterion is assigned an equal weight. Therefore, the weighted standardized decision matrix is the same as the normalized decision matrix in this case.

#### Identify the ideal and anti-ideal solutions

The ideal solution (best performance) and the anti-ideal solution (worst performance) are identified as follows:

- *Ideal solution (maximize)*:
  - Accuracy: 1
  - Precision: 1
  - Recall: 1
  - F1-score: 1
- *Anti-ideal solution (minimize)*:
  - Accuracy: 0
  - Precision: 0
  - Recall: 0
  - F1-score: 0

#### Calculate the Euclidean distances

The Euclidean distance between each alternative and the ideal/anti-ideal solutions is computed using the formula:

2 $$\begin{aligned} D_i^+= & {} \sqrt{\sum _{j=1}^n (r_{ij} - r_j^+)^2} \end{aligned}$$

3 $$\begin{aligned} D_i^-= & {} \sqrt{\sum _{j=1}^n (r_{ij} - r_j^-)^2} \end{aligned}$$

where $D_i^+$ is the distance to the ideal solution, $D_i^-$ is the distance to the anti-ideal solution, $r_{ij}$ is the normalized value of the $i$-th alternative and $j$-th criterion, $r_j^+$ is the ideal value for the $j$-th criterion, and $r_j^-$ is the anti-ideal value for the $j$-th criterion.

Refer to Table 6 for the Euclidean distances.

**Table 6:** Euclidean distances to ideal and anti-ideal solutions

Model	$D_i^+$	$D_i^-$
KNN	0.117	0.882
NN	0.382	0.093
RF	0.382	0.093
SVM-L	0.885	0.140
SVM-R	0.384	0.464
ADA	0.298	0.529
MNB	0.368	0.727
Proposed method	0.016	0.001

#### Compute the relative closeness

The relative closeness of each alternative to the ideal solution is calculated using the formula:

4 $$\begin{aligned} C_i = \frac{D_i^-}{D_i^+ + D_i^-} \end{aligned}$$

Refer to Table 7 for the relative closeness values.

**Table 7:** Relative closeness

Model	Relative closeness
KNN	0.883
NN	0.709
RF	0.709
SVM-L	0.137
SVM-R	0.547
ADA	0.640
MNB	0.664
Proposed method	0.984

#### Rank the alternatives

Finally, the alternatives are ranked based on their relative closeness to the ideal solution. Higher relative closeness values indicate better rankings. The ranking results show that the Proposed Method has the highest relative closeness, indicating it is the best model among the alternatives evaluated.

## Conclusion and future work

This study explores the efficacy of machine learning techniques in predicting stroke occurrences, leveraging Principal Component Analysis (PCA) and a stacking ensemble approach. By optimizing PCA with 16 components, we achieved a notable 98.6% accuracy using a stacked model comprising Random Forest, Decision Tree, and K-Nearest Neighbors (KNN). Our approach not only surpasses traditional models but also highlights the importance of rigorous feature selection and ensemble methods in enhancing predictive performance. These findings underscore the potential of advanced machine learning methodologies in healthcare, particularly for improving stroke risk assessment and patient management strategies.

### Future work

In future work we will incorporate diverse datasets, including genetic, lifestyle, and high-tech imaging data, to strengthen the model’s predictive capabilities. Exploring deep learning techniques tailored for clinical interpretability and further advancements in ensemble learning methodologies offer promising pathways for improvement. To ensure real-world applicability, we propose a multi-phase clinical validation plan, starting with a pilot observational study in three hospitals, enrolling 200 patients. This study will assess the model’s accuracy against established diagnostic methods. Our ultimate goal is comprehensive clinical validation to enhance the model’s credibility and impact on patient care. We seek collaborations with healthcare institutions and funding agencies to support this endeavor, aiming to offer a robust tool for ischemic stroke prediction and patient management.

## Data Availability

The dataset used during the current study is available here.
